# Association of Calcineurin with the COPI Protein Sec28 and the COPII Protein Sec13 Revealed by Quantitative Proteomics

**DOI:** 10.1371/journal.pone.0025280

**Published:** 2011-10-03

**Authors:** Lukasz Kozubowski, J. Will Thompson, Maria E. Cardenas, M. Arthur Moseley, Joseph Heitman

**Affiliations:** 1 Department of Molecular Genetics and Microbiology, Duke University Medical Center, Durham, North Carolina, United States of America; 2 Duke Proteomics Core Facility, Duke University Medical Center, Durham, North Carolina, United States of America; Yonsei University, Republic of Korea

## Abstract

Calcineurin is a calcium-calmodulin-dependent serine/threonine specific protein phosphatase operating in key cellular processes governing responses to extracellular cues. Calcineurin is essential for growth at high temperature and virulence of the human fungal pathogen *Cryptococcus neoformans* but the underlying mechanism is unknown. We performed a mass spectrometry analysis to identify proteins that associate with the calcineurin A catalytic subunit (Cna1) in *C. neoformans* cells grown under non-stress and high temperature stress conditions. A novel prioritization strategy for mass spectrometry data from immunoprecipitation experiments identified putative substrates and proteins potentially operating with calcineurin in common pathways. Cna1 co-purified with proteins involved in membrane trafficking including the COPI component Sec28 and the COPII component Sec13. The association of Cna1 with Sec28 and Sec13 was confirmed by co-immunoprecipitation. Cna1 exhibited a dramatic change in subcellular localization during high temperature stress from diffuse cytoplasmic to ER-associated puncta and the mother-bud neck and co-localized with Sec28 and Sec13.

## Introduction

Calcineurin is a calcium-calmodulin-activated protein phosphatase consisting of two subunits: a 60 kDa catalytic A subunit and a 19 kDa regulatory B subunit, both of which are essential for enzyme activity [1], [2]. Calcineurin is activated by calcium through calmodulin and the calcineurin B regulatory subunit. Calmodulin binds the C-terminal inhibitory region of calcineurin A and relieves inhibition by release of an autoinhibitory domain from the phosphatase active site. Calcineurin effects in yeast and multicellular eukaryotes involve modulation of transcription through dephosphorylation-dependent nuclear translocation of a transcription factor. In *Saccharomyces cerevisiae*, calcineurin dephosphorylates Crz1, triggering its nuclear import and elevating transcription of >160 genes involved in stress responses [3]. In T-cells, calcineurin dephosphorylates the nuclear factor of activated T cell (NFAT) transcription factor, which in turn controls the expression of genes involved in T cell proliferation [4]. Calcineurin is the target of the immunosuppressive drugs cyclosporin A (CsA) and FK506, which block both T cell activation and yeast stress responses.

In addition to transcriptional regulation mediated through the NFAT/Crz1 proteins, calcineurin dephosphorylates diverse substrates that directly influence cell functions. In metazoans, calcineurin has been implicated in numerous processes including cellular trafficking in neuronal cells, apoptosis, and glucose metabolism in muscle cells [5]–[7]. The calcineurin pathway has been implicated in diseases such as Down's syndrome, diabetes, and cardiac hypertrophy [2]. In fungi, a common role of calcineurin is to mediate responses to a variety of stresses, and calcineurin signaling components are well conserved between fungi [8], [9]. However, some stress responses that require calcineurin differ across fungal species. For instance, in *S. cerevisiae* calcineurin is dispensable for growth in standard conditions at both 24°C and 37°C but is essential at high cation concentrations, under cell wall stress, and during prolonged exposure to mating pheromone [3], [10]. In contrast, in *Schizosaccharomyces pombe* calcineurin is essential for cytokinesis and plays an important role in maintaining chloride ion homeostasis [11].

Calcineurin contributes to the virulence of fungal pathogens through mechanisms related to survival in the host, including the ability to grow at high temperature and in serum, and the ability to adhere to host tissues and undergo dimorphic transitions [12], [13]. The human fungal pathogen *Cryptococcus neoformans* requires calcineurin for growth at 37°C, in high levels of CO_2_, alkaline pH, and high cation concentrations [14]. The substrates involved in the calcineurin-mediated stress responses in *Cryptococcus* are largely unknown [15], [16]. Interestingly, no clear functional homologue of the *CRZ1* gene, which encodes the *S. cerevisiae* and *Candida albicans* calcineurin-activated transcription effector, has been identified in *C. neoformans*. It is possible that a different transcription factor is controlled by calcineurin in *C. neoformans* or alternatively, the effects of calcineurin on virulence of *C. neoformans* could be partially or entirely post-transcriptional.

Quantitative mass spectrometry has emerged as a powerful tool for distinguishing specific from non-specific interactions in co-immunoprecipitation experiments (reviewed in [17]. Utilizing a quantitative analysis of affinity-purified samples in comparison to a control with no bait protein, mass spectrometry allows the elucidation of specifically interacting proteins, even in a complex background matrix. This allows for the use of single-step affinity purifications and preserves low-stoichiometry binding partners and weak interactors that are typically lost during standard tandem-affinity purification (TAP) procedures [18], [19]. From these quantitative proteomic experiments, it is possible to identify tens to hundreds of specific protein-protein interactions in a single experiment [20], [21]. This revolution in the ability to identify proteins as part of interactome networks can, in-turn, make data interpretation and functional implication of binding partners challenging. To assist in classification of proteins derived from quantitative affinity purification experiments, we employed an additional quantitative analysis of the cell lysate, with the idea that proteins that were highly abundant in the bait affinity purification, not detected in the control, and also not detected in the lysate are those proteins that are enriched to the highest degree and likely have the highest affinity for the bait. We implemented a decision-tree which can be broadly utilized to classify proteins as “high”, “moderate”, or “low” affinity binders in IP experiments, and deployed this prioritization strategy to interrogate the interactome of calcineurin.

Proteins that associate with the calcineurin catalytic subunit A (Cna1) in non-stressed *C. neoformans* cells and during high temperature stress were identified. A number of proteins that might act with calcineurin in common pathways were established. Association of Cna1 with the COPI component Sec28 and the COPII component Sec13 was confirmed by co-IP. GFP-Cna1 undergoes a dramatic change in subcellular localization during high temperature stress from diffuse cytoplasmic to ER-associated puncta and the mother-bud neck where it co-localizes with Sec28 and Sec13, suggesting that the localization of calcineurin to the ER may represent a key cellular response to temperature stress.

## Results

The approach presented here to identify mechanisms that enable calcineurin to support growth of *C. neoformans* within the host applied mass spectrometry to probe for proteins that associate with calcineurin in a temperature-dependent manner. To isolate calcineurin-associated proteins and examine the localization of calcineurin, the calcineurin catalytic subunit A (Cna1) was tagged with the green fluorescent protein (GFP) at the N-terminus [22]. GFP-fusion chimeras have been recently utilized successfully for purification and identification of interacting proteins by mass spectrometry [23]. The GFP-Cna1 fusion protein was ectopically expressed from the histone H3 promoter in a *cna1*Δ genetic background. Expression of GFP-Cna1 in *cna1*Δ mutant cells restored growth and cellular morphology at 37°C [22], indicating that GFP-Cna1 is functional to operate in pathways that promote growth at high temperatures. A wild type strain lacking GFP-Cna1 was utilized as a negative control for the mass spectrometry analysis. Because GFP alone could not be efficiently expressed in *C. neoformans* unless it contained introns [24], we could not use it as a negative control. However, a recent study has provided evidence that GFP, when used as a tag for protein isolation and subsequent mass spectrometry, has negligible affinity towards other proteins [23].

**Figure 1:**
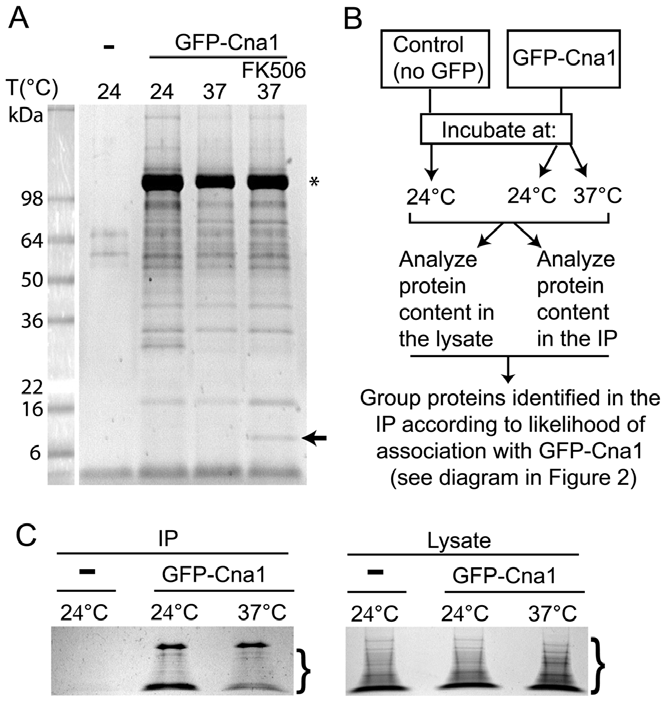
Mass spectrometry (MS) analysis of calcineurin-associated proteins. **A**. A Coomassie blue-stained gel shows that the GFP-Trap resin exhibits a very low affinity towards proteins present in the control lysate, whereas GFP-Cna1 (star) associates with a large number of proteins. An arrow indicates a protein that likely corresponds to FKBP12 in a sample treated with FK506. **B**. Scheme depicting general proteomics approach/strategy. **C**. Coomassie blue-stained gels of the samples that were precipitated using the GFP-Trap resin (left) and the cell lysates analyzed for the protein content as a control (right). For each gel, an area that was excised from the gel for the subsequent MS analysis is depicted (brackets).

**Figure 2:**
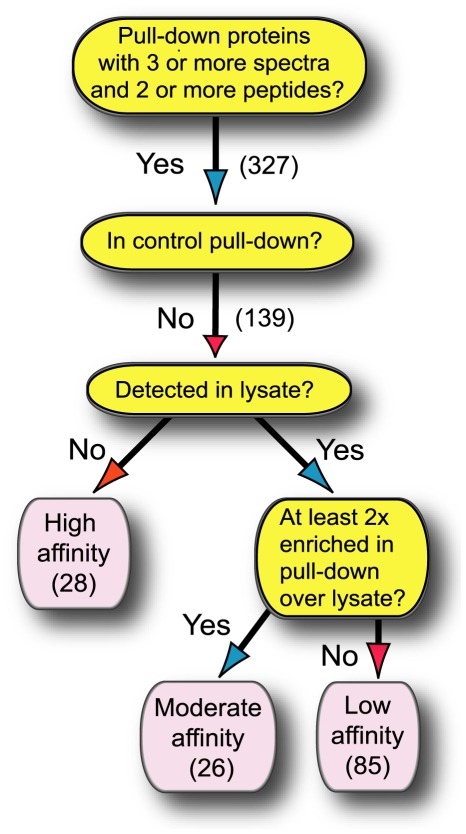
Scheme illustrating the strategy to identify calcineurin-interacting proteins. In principle, a protein that was identified in the immunoprecipitated sample from GFP-Cna1-expressing strain but not from control strain, and which was not detected in the lysate was considered a high affinity interactant. Conversely, a protein that was highly abundant in the lysate and not detected in the purified sample from GFP-Cna1-expressing strain was categorized as non-interacting with Cna1. Other categories reflect the relative amounts of proteins detected in lysates and immunoprecipitated samples.

A variety of proteins were found to interact with GFP-Cna1 when Cna1 was purified via the GFP affinity tag. In contrast, only a very small amount of protein could be isolated from lysates of the control strain (Fig. 1A). To increase the stringency of this approach, and to obtain candidate proteins with a high likelihood of associating with calcineurin, the protein composition of the cell lysates that were used for the affinity purification was also examined by mass spectrometry (Fig. 1B, C). This allowed the evaluation of relative amounts of proteins from cells grown at the two temperatures and determined the basis for grouping proteins detected by mass spectrometry in affinity purified samples according to their likelihood of association with calcineurin (Fig. 2). The tree diagram in Figure 2 depicts the method by which proteins were placed in each category. In principle, a protein identified in the sample affinity purified from the GFP-Cna1-expressing strain but not from the control strain (and that was below the detection limits in the lysate) was considered a tight/significant interaction. On the other hand, a protein that was highly abundant in the lysate and not detected in the purified sample from the GFP-Cna1-expressing strain was categorized as not-interacting with Cna1. Other two categories reflect the relative amounts of the proteins detected in the lysate and compared to the affinity purified samples.

Although this approach allowed identification of a subset of calcineurin-associated proteins with high certainty, it was not designed to identify all calcineurin-associated proteins. Our method for data evaluation did not allow for the asserting whether proteins that are highly abundant associate with calcineurin. In principle, a protein could have been detected as associated with calcineurin because its levels in the lysate were very high resulting in a false positive interaction. On the other hand, such a highly abundant protein may actually interact with calcineurin. A good example is Hsp90, which is highly abundant and was detected as interacting with calcineurin in our analysis. We evaluated proteins from gel slices excised from the area of the gel just below the robust signal corresponding to GFP-Cna1 (Fig. 1C), with molecular masses below that of GFP-Cna1 (∼100 kDa). Consequently, identification of proteins larger than 100 kDa was unlikely, except for proteins that underwent degradation. Analysis of ORFs from *S. cerevisiae* indicates that ∼85% of proteins encoded by the genome are below 100 kDa [25]. Thus, our analysis likely covered a majority of the proteins expressed in *C. neoformans*. Despite these limitations, this methodology allowed the identification of a number of novel proteins that likely associate with calcineurin in temperature-dependent and -independent manners.

### Proteins associated with Cna1

A rigorous method was employed to determine the proteins that are the most likely to be associated with calcineurin (Fig. 2). 327 proteins (∼4% of the predicted protein coding genes) were identified that met the first criterium of 3 or more spectra and 2 or more peptides detected. After eliminating proteins identified in the control pull-down, the number of potential interactions was 139. This group was divided into three categories. First, the elimination of proteins that were detected in the lysate allowed identification of 28 proteins that most likely associate with calcineurin designated as high affinity interactions. Second, 26 proteins that were at least 2 times enriched in the pull-down as compared to the lysate were classified as moderate affinity interactions. Third, 85 proteins that were not significantly enriched in the pull-down were called low affinity interactions. Table S1 includes proteins that were identified using the method described in Figure 2 and grouped according to common cellular function or location.

Notably, the calcineurin regulatory subunit Cnb1 and calcipressin Cbp1, both previously characterized in *Cryptococcus* [26], [27] and both conserved Cna1-interacting proteins, bound with high affinity to GFP-Cna1. This finding confirmed that the method indeed allowed identification of calcineurin-associated proteins. Interestingly calmodulin was not detected either in the lysates or in the immunoprecipitated samples. Inability to detect calmodulin in the lysates may result from an overall low abundance. However, lack of detection in the immunoprecipitated samples is puzzling. Further studies are needed to explain this unexpected result.

The most prominent functional groups included mitochondrial proteins, proteins involved in cell metabolism, membrane fusion and cellular trafficking components, proteins related to the translation machinery, as well as heat shock proteins and their regulators (Table S1). Several proteins exhibited differential association with calcineurin at 24°C and 37°C, as discussed below.

### GFP-Cna1 associates with the COPI component Sec28 and the COPII component Sec13 *in vivo*

Among the proteins that showed a significant association with calcineurin were the COPI component Sec28 and the COPII component Sec13. In *S. cerevisiae*, Sec28 is an epsilon-COP subunit of the coatomer that regulates retrograde Golgi-to-ER protein traffic [28]. Sec13 is a component of the Sec13-Sec31 complex of the COPII vesicle coat required for vesicle formation during ER to Golgi transport [29]. Sec13 is also a component of the Nup84 sub-complex required for organization of the nuclear pore complex [30].

**Figure 3:**
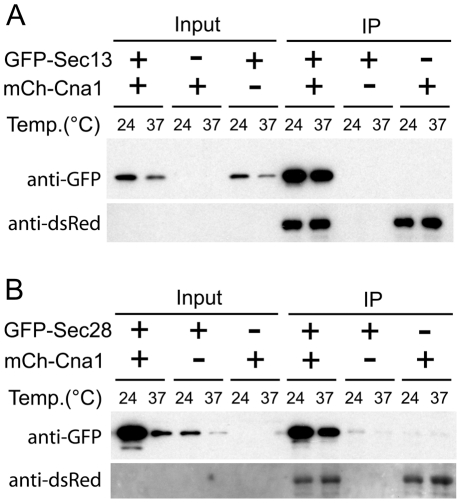
Sec13 and Sec28 co-precipitate with calcineurin A from cell lysates. **A**. A co-IP of the GFP-Sec13 with the mCherry-Cna1 using the RFP-Trap resin. A strain that expresses only GFP-Sec13 and a strain that expresses only mCherry-Cna1 served as negative controls. The membrane was initially probed with an anti-GFP antibody, stripped and subsequently probed with an anti-dsRed antibody to detect the precipitated mCherry-Cna1. **B**. A co-IP of GFP-Sec28 with mCherry-Cna1 using the RFP-Trap resin. A strain that expresses only GFP-Sec28 and a strain that expresses mCherry-Cna1 but not GFP-Sec28 served as negative controls. The membrane was probed with an anti-GFP antibody and subsequently probed with an anti-dsRed antibody to detect the precipitated mCherry-Cna1.

In another study we have shown that upon high temperature stress calcineurin re-localizes to cytoplasmic puncta likely associated with the ER [22]. We hypothesized that Cna1 localization to these puncta during high temperature stress may result from association with Sec13 and Sec28. To validate the mass spectrometry data, and confirm that Cna1 associates with Sec13 and Sec28, GFP-Sec13 and GFP-Sec28 were co-expressed with mCherry-Cna1 and the mCherry-Cna1 was precipitated with RFP-Trap resin. The mCherry-Cna1 chimera was expressed from a chromosomally integrated transgene expressed from the *GPD1* promoter. Both GFP-Sec13 (Fig. 3A) and GFP-Sec28 (Fig. 3B) were efficiently co-precipitated from cells that also expressed mCherry-Cna1, whereas neither of the GFP chimeras showed affinity to the RFP-Trap resin. Probing with an anti-dsRed antibody confirmed an efficient precipitation of the mCherry-Cna1, although this antibody did not detect the protein in input samples (presumably because of low protein abundance). Notably, the extent of association of Cna1 with Sec13 or Sec28 was independent of growth temperature, in accord with the mass spectrometry data.

### GFP-Cna1 co-localizes with the COPI component Sec28 and the COPII component Sec13 during high temperature stress

**Figure 4:**
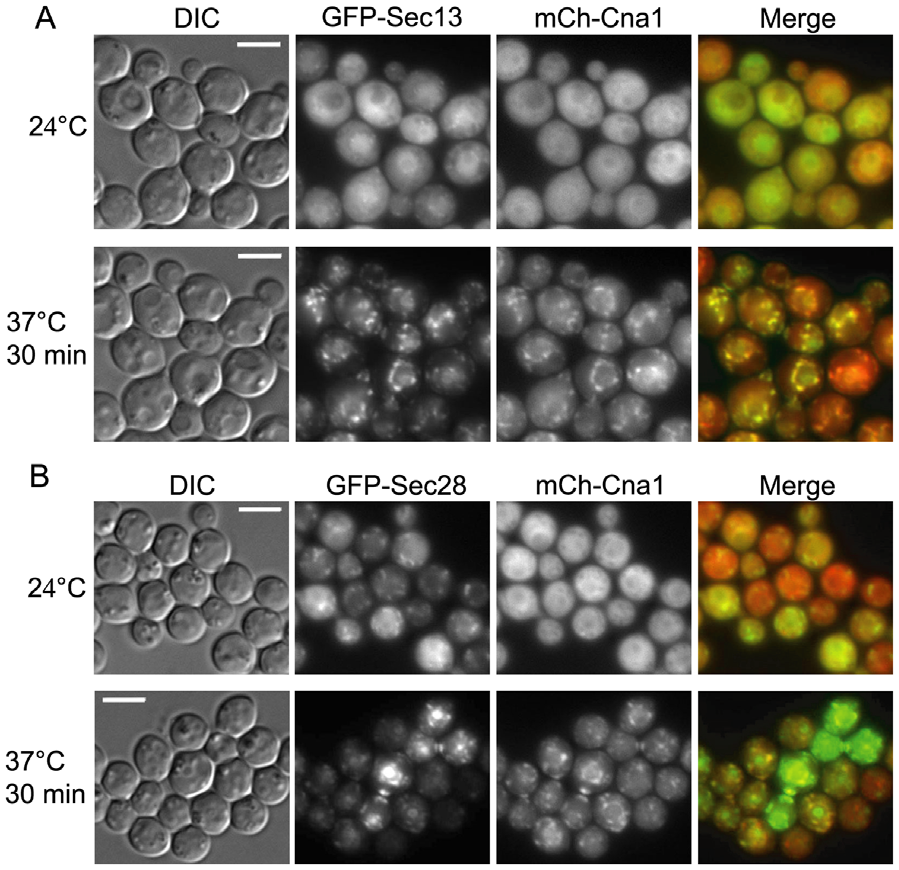
mCherry-Cna1 co-localizes with (A) GFP-Sec13 and with (B) GFP-Sec28 during a rapid temperature shift to 37°C. Cells were initially grown at 24°C, and the slide containing the cells grown on an agarose patch was placed on a heating block preset to 37°C. The cells were imaged after 30 minutes of incubation at 37°C. Scale bar equals 5 µm.

The association of Cna1 with Sec13 and Sec28 at both 24°C and 37°C prompted us to examine the localization of GFP-Sec13 or GFP-Sec28 together with mCherry-Cna1 at 24°C and after the temperature shift to 37°C (Fig. 4). The mCherry-Cna1 chimera was diffusely cytoplasmic at 24°C and its localization shifted to cytoplasmic puncta around the nucleus and at the mother-bud neck at 37°C (Fig. 4). This was consistent with our other study, which showed that during thermal stress, Cna1 localizes to puncta surrounding the nucleus [22]. At 24°C GFP-Sec13 was cytoplasmic and also enriched in the nucleus and puncta that likely represent exit sites within the ER (Fig. 4A) [31], [32]. Under these conditions, GFP-Sec28 was also cytoplasmic and localized to puncta or less structured membrane-like signals, consistent with its association with Golgi membranes (Fig. 4B). Upon a rapid temperature shift to 37°C, both GFP-Sec13 and GFP-Sec28 largely co-localized with mCherry-Cna1 (Fig. 4). This co-localization included puncta surrounding the nucleus as well as at the mother-bud neck and strikingly resembled the appearance of general ER membranes in other yeasts [32]. In addition, our recent study shows that Cna1 co-localizes with a P-body marker Dcp1 that associates with the ER at 37°C [22]. These data indirectly suggest that although Sec13 and Sec28 occupy different sites at 24°C, during a rapid temperature change to 37°C they co-localize to the regions likely corresponding to the general ER.

Given the association of Cna1 with Sec13 and Sec28, either or both could be substrates of calcineurin. However, treatment of cells expressing the chimeric proteins with the calcineurin inhibitor FK506 did not significantly change the migration pattern of the GFP-Sec13 or GFP-Sec28 proteins in 1D-PAGE analysis, nor did it influence the localization of these proteins at 24°C or 37°C (data not shown). These observations suggest that Sec13 and Sec28 may not be direct substrates of calcineurin, although a modest electrophoretic mobility change after dephosphorylation by calcineurin might not be detected by this approach, or the protein may be a substrate whose mobility is not influenced by phosphorylation. Alternatively, they could be directly or indirectly regulated by calcineurin in a manner that does not involve the catalytic activity of calcineurin.

## Discussion

The aim of this study was to identify proteins that associate with the calcineurin catalytic subunit Cna1 and that may be relevant to the thermal stress response mediated by calcineurin in *C. neoformans*. A strong interaction was detected between Cna1 and the calcineurin regulatory subunit Cnb1 and the calcipressin Cbp1, which validates this analysis.

Our study provided for the first time an extensive list of proteins that likely associate with calcineurin (Table S1). The only analysis that we are aware of, which utilized mass spectrometry to identify calcineurin partners (in addition to high throughput studies) is a work by Tokheim and Martin [33]. In this study calcineurin was immunoprecipitated from mouse hearts and mitochondrial proteins were identified as interacting partners, specifically Mn-superoxide dismutase (SOD), aconitase (ACN), and malate dehydrogenase (MDH). Our analysis also revealed an association of calcineurin with ACN and MDH and a number of mitochondria related proteins suggesting that calcineurin regulates mitochondrial processes. Interestingly however, no specific co-localization was detected between GFP-Cna1 and mitochondria in *C. neoformans* [22].

Importantly, our study identified several novel calcineurin-associated proteins previously implicated in processes related to calcineurin function. Among this category was the alpha-trehalose-phosphate synthase, Tps1, responsible for synthesis of trehalose, a key molecule required for stress survival in fungi [34]. Tps1 is necessary for *Cryptococcus* growth at 37°C, similar to calcineurin [35]. Some temperature-sensitive mutants require calcineurin for growth at 24°C (are sensitive to FK506) implicating the mutated gene and calcineurin in parallel pathways. On the other hand, if the temperature-sensitive mutant is not sensitive to calcineurin inhibition at 24°C, the mutated gene likely operates with calcineurin in a common pathway. A *tps1*Δ mutant grows on media supplemented with the calcineurin inhibitor FK506 at 24°C, suggesting that Tps1 and Cna1 may operate in a common pathway (our unpublished results). In addition, the *tps1*Δ mutant cells exhibit a cytokinesis defect at 37°C similar to that of the *cna1Δ* mutant (our unpublished results). The Tps1 enzyme is also thought to be regulated by reversible protein phosphorylation/dephosphorylation in a partially calcium-dependent manner [36], [37]. It will be interesting to probe whether Tps1 is indeed binding to, and perhaps being dephosphorylated by calcineurin and whether calcineurin regulates trehalose levels during stress response.

In addition to Tps1, this study identified several other metabolic enzymes potentially interacting with calcineurin. Notably, enzymes functioning in glycolytic pathway were detected, including glycerol-3-phosphate dehydrogenase. Calcineurin regulates glucose metabolism in skeletal muscle [7]. Calcineurin is necessary for *C. albicans* to survive calcium stress in serum [38] and a number of classes of *C. albicans* genes are upregulated in serum, including genes involved in glycolysis [39]. This suggests a possible role of calcineurin in the regulation of carbohydrate metabolism in fungi.

Another metabolic enzyme and a putative substrate of calcineurin identified in this study is NADP^+^-dependent glutamate dehydrogenase (NADP-GDH). Interestingly, the levels and phosphorylation state of NADP-GDH are associated with the dimorphic transition between yeast and hyphae in fungi [40], [41]. Calcineurin-controlled morphogenic transitions have been documented in major human and plant fungal pathogens including *C. neoformans*, *Aspergillus fumigatus*, *Magnaporthe oryzae*, and *Ustilago maydis* (recently reviewed in [42]). Thus, it is plausible that one of the mechanisms by which calcineurin contributes to morphological conversions is modification of the phosphorylation state of NADP-GDH.

Recently, Kmetzsch et al. has established that Vcx1 a vacuolar calcium exchanger is involved in calcineurin-dependent Ca^2+^ tolerance, acts in the Ca^2+^-calcineurin signaling pathway in *C. neoformans*, and influences the relative intracellular calcium concentration [43]. Our analysis did not reveal an association of Cna1 with Vcx1 or any other calcium transporters suggesting that calcineurin may not physically interact with these proteins. This however, does not exclude the possibility of an indirect regulation.

Interestingly, no transcription factor was identified as interacting with Cna1 in our study. This could simply result from a weak or transient nature of such an interaction, or from the relatively low abundance of transcription factors. This could also result from a similar or greater size of the transcription factor to that of GFP-Cna1, a previously described limitation of this approach. Potential strategies to overcome these limitations in future work are to cross-link the proteins prior to immunoprecipitation, or perform cellular fractionation to isolate the nuclear fraction. It is also possible that calcineurin mediated transcriptional regulation during temperature stress in *Cryptococcus* does not represent the major response involving calcineurin. Instead, calcineurin may act primarily by associating with and perhaps dephosphorylating other targets.

Among the proteins identified in this study is the homolog of Slm1, a calcineurin substrate during heat stress responses in *S. cerevisiae* [44]. A ∼36-fold increase in binding of Slm1 to calcineurin upon heat stress suggests that calcineurin-Slm1 regulation is conserved in the Basidiomycota. Slm1 proteins are subject to regulation by multiple signals including sphingolipid signaling [45]. Sphingolipids mediate formation of P-bodies that are ER-associated sites of mRNA processing [46]. Our data suggest that Cna1 localizes to ER membranes during heat stress. Therefore, it is plausible that calcineurin regulates Slm1 at the ER membrane during heat stress, although a reciprocal relationship between Slm1 and calcineurin is also possible. *S. cerevisiae* Slm1 and Csg2 (an enzyme required for production of the sphingolipid mannosylinositolphosphoceramide) cooperate to negatively regulate cellular calcineurin activity [47]. On the other hand, *S. cerevisiae* calcineurin is not required for growth at 37°C. Interestingly, whereas the *S. cerevisaie slm1*Δ mutant exhibits a defect in the heat-induced levels of inositol phosphorylceramide (IPC) one of the downstream products of shingolipid metabolism [47], a decrease in expression of IPC synthase in *C. neoformans* does not result in temperature-sensitive growth [48]. Future studies will be directed to explore the relationship between calcineurin, Slm1, and sphingolipid metabolism and their involvement in heat stress.

In another study we found that calcineurin co-localizes with P-bodies and stress granules in cytoplasmic puncta at 37°C [22]. P-bodies are cytoplasmic foci where mRNA species are stored and degraded, whereas stress granules are formed specifically in response to stress and represent sites where the synthesis of “housekeeping” proteins is inhibited to prioritize the synthesis of proteins specific to stress response [49]. A role for calcineurin in the regulation of translation has been suggested in other eukaryotes but the precise mechanisms remain elusive. Although no typical components of P-bodies or stress granules were identified in the study presented here, a number of proteins related to translation were identified as interacting with calcineurin. One protein identified here as associated with Cna1, Tpa1, was previously shown to physically interact with P-body constituent Pab1 and represents possible link between Cna1 and sites of mRNA processing [50]. Particularly intriguing is a significant decrease of binding between Cna1 and eIF2β at 37°C potentially related to regulation of translation. Therefore, it will be of interest to further elucidate possible mechanisms of calcineurin-mediated translational regulation during stress.

The demonstration of an association between calcineurin and COPI and COPII complexes is unprecedented. Our data indicate that Cna1 associates with Sec28 and Sec13 irrespective of growth temperature. Interestingly, although Sec28 was classified in our analysis as a high affinity interactant and Sec13 as a moderate affinity interactant, this difference was not apparent in the immunoprecipitation experiment. This discrepancy stems from the fact that Sec13 but not Sec28 was detected in the cell lysate. This indicates that we should be cautious when drawing conclusions about the strength of interaction with Cna1 based on the mass spectrometry results and such conclusions should always be based on additional complementary studies. The localization of Sec13 and Sec28 does not depend on calcineurin activity because it was not affected by treatment with the calcineurin inhibitor FK506. The COPII vesicles containing Sec13 assemble at the transitional ER (tER) that in some cells can be directly adjacent to the *cis*-face of Golgi stacks, or alternatively, they may be dispersed throughout the cytoplasm with no physical contact with the Golgi [32]. Although the physiological significance of the Cna1 association with Sec28 and Sec13 remains to be elucidated, our findings indicate that upon high temperature stress, Sec13 and Sec28 likely co-localize, indicative of a fusion between the Golgi and ER membranes. Spatial separation of the Golgi and ER is crucial for cell physiology [51], indicating that the putative co-localization of Sec13 and Sec28 may represent an abnormal state.

Interestingly, among the “weak interactants” a homologue of the Arf1 small monomeric GTPase was identified. Although, according to our criteria, this interaction would not be considered significant due to a high abundance of Arf1 in the cell, it is consistent with the identification of the COPI component Sec28 and suggests a possible regulation of Sec28 by calcineurin.

One possible function of calcineurin during stress response is regulation of membrane trafficking. Calcineurin may contribute to the return to the “normal” state after the initial temperature stress [52]. In *S. cerevisiae*, several mutants defective in vacuolar function exhibit hypersensitivity to FK506 and require calcineurin for viability [53]–[55]. Some of these mutants require calcineurin for its ability to act at the transcriptional level through Crz1 and promote expression of the vacuolar Ca^2+^-ATPase Pmc1, whereas others may involve post-transcriptional roles of calcineurin [55]. Although the role of calcineurin in vacuole function remains rather speculative, our mass spectrometry and microscopy data suggest a connection between calcineurin and COPI and COPII complexes, as well as a possible association with the dynamin-like GTPase Vps1. In *S. cerevisiae*, Vps1 is required for the formation of transport vesicles in the Golgi, possibly involving scission [56]. Our study also identified small COPII coat GTPase SAR1 and a coatomer subunit gamma, Sec21 underscoring possible role of calcineurin in membrane trafficking.

In metazoans, calcineurin regulates vesicle transport by dephosphorylating several relevant proteins, including dynamin, amphiphysin, and synaptojanin. Calcineurin regulates both clathrin-dependent and independent endocytosis in synapses [57] and promotes endocytosis in *C. elegans* through dynamin [58]. Thus, the role of calcineurin in membrane trafficking is likely conserved between fungi and metazoans.

**Figure 5:**
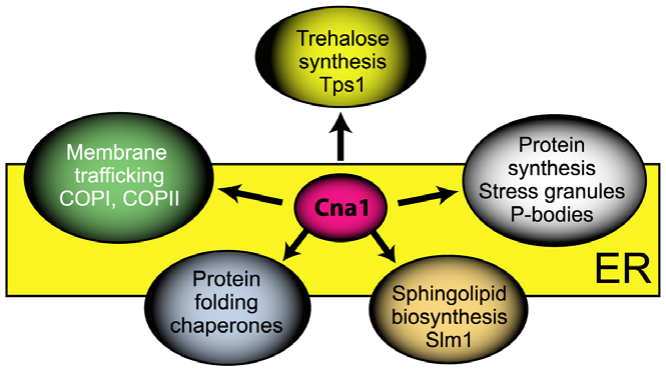
A hypothetical model showing multiple processes controlled by calcineurin during thermal stress in *C. neoformans*. The model is based on findings presented in this study (the association of calcineurin catalytic subunit (Cna1) with trehalose synthase Tps1, Slm1, and ER chaperones, a putative role of Cna1 in vacuole morphology, and localization of Cna1 to the ER during thermal stress), our other studies (co-localization of Cna1 with ER-associated sites of mRNA processing, P-bodies and stress granules during stress), and the work of others.

In summary, our study demonstrates an association of calcineurin A with a number of intriguing proteins in *C. neoformans*. Two lines of evidence suggest that during stress calcineurin associates with ER membranes. First, Cna1 co-localizes with COPI and COPII components during heat stress. Second, stress-induced localization of Cna1 overlaps with P-bodies and stress granules that form ER-associated foci [22]. Association of Cna1 with ER is also supported by identification of Yop1 as a potential Cna1-interacting protein. Yop1 is a tubule-shaping protein that stabilizes membrane curvature at all peripheral ER domains in *S. cerevisiae* [59]. What could be the ER-related functions of calcineurin during thermal stress? Based on this study, our recent findings [22], and work of others, we propose that during thermal stress calcineurin associates with ER membranes and controls multiple aspects of cellular stress response by mechanisms that do not involve transcriptional regulation (Fig. 5). The exact mechanisms of calcineurin contribution to stress survival remain elusive. Calcineurin could dephosphorylate target proteins at the ER, or alternatively, act as a scaffold. Some proteins may tether calcineurin to the ER and bring the phosphatase to the proximity of its target substrates. Our recent study shows that Cna1 associates with the cytoplasmic puncta in response to other stresses in addition to temperature stress [22] and calcineurin activity towards some substrates may be stress-specific. Studies using this genetically amenable model system could contribute to understanding roles of calcineurin in processes related to cell stress. We believe this study will also facilitate elucidation of calcineurin-dependent processes in other organisms including humans.

## Materials and Methods

### Strains, media, and growth conditions

*C. neoformans* strains and plasmids used in this study are listed in Table 1. All media were prepared as described previously [60]. To generate strain LK281, plasmid pLKB60 [22] encoding mCherry-Cna1 was integrated into strain H99 by biolistic transformation [61]. Strain LK281 was utilized to construct strains LK287 and LK288 by integrating plasmids pLKB78 and pLKB79.

**Table 1:** List of strains and plasmids used in this study.

Strain	Genotype	Source/Reference
H99	Alpha	[62]
KN99	**A**	[63]
KK8	**a** *cna1::NEO*	[64]
LK214	a *cna1::NEO GFP-CNA1:NAT*	[22]
LK281	α *mCH-CNA1:NEO*	This study
LK287	α *GFP-SEC28:NAT mCH-CNA1:NEO*	This study
LK288	α *GFP-SEC13:NAT mCH-CNA1:NEO*	This study
LK289	α *GFP-DCP1:NAT mCH-CNA1:NEO*	[22]
LK291	**a** *GFP-SEC28:NAT*	This study
LK292	**a** *GFP-SEC13:NAT*	This study

Plasmid	Genotype	Source/Reference
pLKB39	*GFP-CNA1:NAT*	[22]
pLKB60	*mCH-CNA1:NEO*	[22]
pLKB78	*GFP-SEC28:NAT*	This study
pLKB79	*GFP-SEC13:NAT*	This study

### Generating fluorescent protein chimeras

All strains expressing N-terminally tagged fluorescent proteins were made by ectopic integration of a respective plasmid encoding the fluorescent chimera expressed from either the histone H3 promoter (GFP-Cna1, GFP-Sec13, GFP-Sec28) or the *GPD1* promoter (mCherry-Cna1). Integrations were performed by biolistic transformation [61]. Positive clones were screened based on growth on media containing the appropriate drug and confirmed by examining the fluorescent signal. Plasmid pCN19 (kindly provided by Connie Nichols and Andy Alspaugh, Duke University) was employed to make GFP chimeras and the PCR products of a respective gene were cleaved with BamHI. To amplify *SEC13* ORF (CNAG_ 04194) to generate pLKB79, primers JOHE26005 (CAAGGATCCGCTATGGTATGTTCCGGAACCATATAAC ) and JOHE26006 (CTAGGATCCATACAGGTCGCAGCAACATGC) were used. To amplify the *SEC28* ORF (CNAG_ 01211) to generate pLKB78, primers JOHE25997 (CAAGGATCCGCTATGGAGGCAGACCCCTTATAC) and JOHE25998 (CTAGGATCCCGAAGTGAGTCGTATCTATC) were used.

### Sample preparation for mass spectrometry

Cell cultures of the strain expressing GFP-Cna1 (strain LK214) and a control strain (KN99**a**) were grown in YPD at 24°C to an optical density OD_600_ ∼0.7. Next, the culture was divided into two 500 ml cultures, and one was incubated at 37°C and the other at 24°C. At the same time, 500 ml of the control culture was incubated at 24°C. After 1 hour of incubation, cells were rapidly chilled using dry ice, spun at 4°C, washed with lysis buffer containing protease inhibitors, and the pellets were stored at −80°C. Cells were lysed in 30 ml of lysis buffer (10 mM Tris/Cl pH = 7.5, 150 mM NaCl, 0.5 mM EDTA; supplemented with protease inhibitor tablets (Roche) and 1 mM PMSF) using a French press. For the analysis of proteins associated with GFP-Cna1, ∼30 ml of cell lysate (containing ∼90 mg protein) was incubated for 2 hours at 4°C with 30 µl of GFP-Trap slurry prepared according to the manufacturer's instructions (Chromotek Gmbh). GFP-Trap beads were washed 3× with the wash buffer (10 mM Tris/Cl pH = 7.5, 350 mM NaCl, 0.5 mM EDTA; supplemented with protease inhibitor tablets (Roche), 1 mM PMSF, and 1 mM DTT), resuspended in 30 µl of electrophoresis sample buffer (Santa Cruz Biotech, Santa Cruz, CA), boiled for 10 min, and the supernatant was resolved on an SDS PAGE gel. The gel was stained with Coomassie blue and the region between approximately 6 and 100 kDa for each lane was cut into 6∼3 mm thick gel slices, as indicated in Figure 1. Slices that contained GFP-Cna1 (just above ∼100 kDa) were not included in the analysis.

To ascertain the relative protein expression levels in the lysate as a control, we analyzed the composition of the lysates within the same molecular weight range as that of the pulldown. Aliquots of the lysates for GFP-Cna1-expressing cells grown at 24°C and 37°C, containing ∼15 µg of protein, were boiled with the SDS loading dye for 5 min and separated on an SDS PAGE gel to a maximum separation of ∼1.5 cm. The gel was stained with Coomassie blue and the target molecular weight range was cut into 5 ∼3 mm gel slices as indicated in Figure 1.

The excised gel bands were destained and the proteins in the bands were digested with trypsin according to the “*In-Gel Tryptic Digestion Protocol (Abbreviated)*” available at (http://www.genome.duke.edu/cores/proteomics/sample-preparation/). Briefly, bands were destained with 1∶1 MeCN/water, then dehydrated in MeCN and swelled in 50 mM ammonium bicarbonate containing 10 ng/µl trypsin. Digestion was carried out overnight at 37°C, and digestion was quenched and peptides were extracted using 0.1% v/v TFA in 1∶1 MeCN/water. Samples were dried and reconstituted in 10 µl 1/2/97 v/v/v TFA/MeCN/water for mass spectrometry analysis.

### Quantitative mass spectrometry

Mass spectrometry data collection for all gel band samples (both pulldown and lysate) was performed in an equivalent manner. Five microliters of each sample was injected onto a 75 µ×250 mm BEH C18 column (Waters) and separated using a gradient of 5 to 40% (vol/vol) acetonitrile with 0.1% (vol/vol) formic acid, with a flow rate of 0.3 µl/min for 90 minutes on a nanoAcquity liquid chromatograph (Waters). The eluent was introduced to an LTQ-Orbitrap hybrid mass spectrometer (Thermo) using a nanoelectrospray interface. The Orbitrap MS/MS method utilized CID fragmentation for peptide identification, with both precursor and product ions being measured in the Orbitrap. Briefly, the precursor scan method utilized profile mode and 60,000 resolution, with AGC target of 1e6 and 1 microscan. MS/MS acquisition was performed on the top three precursor ions above a 5000-count threshold, using collisionally induced dissociation (CID) with a 3 Da isolation window, normalized collision energy of 35% and 1 microscan. Product ion spectra were collected in profile mode with a resolution of 7500 and AGC target setting of 2e5. Dynamic exclusion settings were the following: repeat count was 3, repeat duration was 30 sec, exclusion list was 250, and exclusion time was 120 sec.

For qualitative identifications and spectral-counting quantitation, Mascot Distiller v2.2 (Matrix Sciences, Inc.) was utilized to generate mascot-searchable files from .raw mass spectrometry data. Database searching was performed with Mascot v2.2 against the *C. neoformans* H99 database (*Cryptococcus neoformans var. grubii* H99 Sequencing Project, Broad Institute of Harvard and MIT, http://www.broadinstitute.org/, April 2011), and the following search parameters: 10 ppm precursor and 0.02 Da product ion mass accuracy, tryptic enzyme specificity, a maximum of two missed cleavages, and oxidized (M) and deamidated (NQ) as variable modifications. Data was curated using PeptideProphet and ProteinProphet algorithms in Scaffold v3.0 (www.proteomesoftware.com) [Keller, A et al Anal. Chem. 2002;74(20):5383–92, Nesvizhskii, A et al Anal Chem. 2003 Sep 1;75(17):4646–58]. Spectral counting data analysis was performed utilizing the spectrum counting report from Scaffold v3.0. This data has been made publicly available at the following link: https://discovery.genome.duke.edu/express/resources/2122/Kozubowski_MSMS_Supplementarydata_April2011.sf3.

In order to perform quantitative analysis between the very similar ambient v 37C pulldown conditions, the data was imported into Rosetta Elucidator v3.3 (Rosetta Biosoftware, Inc.) software for robust label-free under the curve (AUC) quantitation. Importantly, this approach was only used to determine for each binding category whether or not there was differential binding as a function of temperature. LC-MS feature quantitation and data alignment between samples was performed within Elucidator v3.3, allowing quantitation of all peptides in both 37C and 24C pulldown samples. Peak annotation was performed using database searching (as described above) at a peptide false-discovery rate of 1%. Protein quantitation, and associated fold-changes and p-values for each sample were then calculated as a sum of the peak areas of all peptides. Values for proteins in each binding category are reported in Table S1, using a fold-change of 2 and p<0.01 to denote statistical significance.

### In vivo co-immunoprecipitation

Cell cultures (100 ml) were grown in YPD at 24°C to an optical density OD_600_∼0.7. Next, each culture was divided into two 50 ml cultures, one of which was incubated at 37°C and the other at 24°C. After 1 hour of incubation, cells were rapidly chilled using dry ice, spun at 4°C, washed with lysis buffer containing protease inhibitors, and the pellets were stored at −80°C. Cells were lysed in 1 ml of lysis buffer (10 mM Tris/Cl pH 7.5, 150 mM NaCl, 0.5 mM EDTA; supplemented with protease inhibitor tablets (Roche), 1 mM PMSF and 1 mM DTT) using a Mini Beadbeater (Biospec Products). To precipitate mCherry-Cna1, 0.3 ml of cell lysate was incubated for 2 hours at 4°C with 10 µl of RFP-Trap slurry prepared according to the manufacturer's instructions (Chromotek Gmbh). RFP-Trap beads were washed 3× with the wash buffer (10 mM Tris/Cl pH 7.5, 350 mM NaCl, 0.5 mM EDTA; supplemented with protease inhibitor tablets (Roche), 1 mM PMSF, and 1 mM DTT), resuspended in 15 ul of electrophoresis sample buffer (Santa Cruz Biotech, Santa Cruz, CA), boiled for 10 min and the supernatant was resolved on an SDS PAGE gel. To detect GFP chimeras, an anti-GFP polyclonal antibody (Santa Cruz Biotech, Santa Cruz, CA) was used at a 1∶1000 dilution. To detect mCherry chimeras an anti-dsRed polyclonal antibody (Santa Cruz Biotech, Santa Cruz, CA) was used at a 1∶1000 dilution.

### Microscopy

For imaging yeast cells, ∼0.5 µl of cell suspension was placed on a thin 2% agarose patch on the slide and covered with a cover slip. Brightfield, differential interference microscopy (DIC), and fluorescence images were captured with a Zeiss Axioscope equipped with an ORCA cooled charge-coupled device camera (Hamamatsu, Bridgewater, NJ), and interfaced with MetaMorph software (Universal Imaging, Silver Spring, MD). Images were processed using Photoshop (Adobe Systems, San Jose, CA).

## Supporting Information

Table S1 **Proteins identified as associated with Cna1 and grouped according to cellular function and/or location.** A fold change in binding at 37°C (a negative value indicates an increase in binding at 37°C) and corresponding P-values are indicated.
(PDF)
