# Supplementary material for: Association of Calcineurin with the COPI Protein Sec28 and the COPII Protein Sec13 Revealed by Quantitative Proteomics
Source: PLoS One. 2011 Oct 3;6(10):e25280. doi: 10.1371/journal.pone.0025280 (PMC3184950; doi:10.1371/journal.pone.0025280)
Supplement: Table S1 — Proteins identified as associated with Cna1 and grouped according to cellular function and/or location. A fold change in binding at 37°C (a negative value indicates an increase in binding at 37°C) and corresponding P-values are indicated. (PDF) [file pone.0025280.s001.pdf]

Table S1. Proteins identified as associated with Cna1 and grouped according to cellular function/location.

				Affinity Class			24°C vs 37°C Quantitation		Stat. signif.
CNAG *	Description	kDa	Peptide count	H	M	L	Fold change**	P-value	
00888	calcium-dependent protein phosphatase Cnb1	20	11	X			1.4	6.4E-04	
00802	calcineurin-binding protein, calcipressin Cbp1	27	11	X			-3.2	1.5E-24	X
	**Proteins related to mitochondria**								
00162	alternative oxidase	44	3	X			-6.6	3.8E-01	
03824	Phosphate transport protein MIR1	34	3	X			-6.2	3.6E-01	
03106	54S ribosomal protein L35,	43	2	X			-1.0	9.6E-01	
04709	ribosomal protein L23	40	2	X			4.4	2.8E-01	
05809	Ferrochelatase Hem15	45	2	X			-1.5	7.1E-01	
00499	carnitine/acyl carnitine carrier	33	2	X			6.8	5.4E-01	
03931	import receptor subunit Tom40	41	5		X		1.5	7.4E-02	
01688	ATP-dependent peptidase	77	3		X		-1.0	9.8E-01	
00747	succinate-CoA ligase	45	10		X		-1.6	9.8E-02	
01639	elongation factor G 1	90	5		X		-2.3	1.3E-02	
07810	mitofilin	80	8			X	-2.6	9.4E-06	X
05909	electron transporter	35	4			X	1.4	4.9E-01	
02315	ubiquinol-cytochrome c reductase	30	4			X	-2.3	2.4E-01	
00533	inner membrane translocase subunit TIM50	57	3			X	-1.4	4.5E-01	
05132	cytochrome c oxidase	17	2			X	-1.9	5.5E-01	
03225	malate dehydrogenase	36	6			X	6.9	8.5E-04	X
00237	3-isopropylmalate dehydratase	83	5			X	-1.3	3.5E-01	
03596	dihydrolipoamide succinyltransferase	49	5			X	6.3	2.7E-08	X
07908	aconitate hydratase	84	2			X	-1.8	2.4E-01	
07660	pyruvate dehydrogenase e1 component alpha	46	2			X	1.0	9.8E-01	
05031	succinyl-CoA:3-ketoacid-coenzyme A transferase	60	2			X	2.8	3.5E-01	
01586	ATP synthase	26	3			X	3.1	3.5E-01	
	**Amino acid metabolism**								
06370	branched-chain-amino-acid aminotransferase	47	4	X			2.0	1.6E-01	
00622	acetylornithine transaminase	50	7		X		1.4	4.6E-01	
04990	glutamate-5-semialdehyde dehydrogenase	49	6		X		5.1	3.9E-03	X
05398	phosphoserine transaminase	45	5		X		4.2	4.3E-02	
04951	phospho-2-dehydro-3-deoxyheptonate aldolase	40	4		X		1.0	9.9E-01	
02686	cystathionine beta-lyase	98	2		X		-1.9	5.2E-01	
05602	1-pyrroline-5-carboxylate dehydrogenase	60	18			X	32.5	0.0E+0	X
03457	arginine-tRNA ligase	70	8			X	10.8	1.2E-11	X
06755	threonine-tRNA ligase	84	7			X	2.0	8.6E-02	
04604	tryptophan-tRNA ligase	68	3			X	-2.4	2.7E-01	
01305	methionyl-tRNA synthetase	81	3			X	1.0	9.7E-01	
02763	phenylalanyl-tRNA synthetase	71	7			X	1.4	4.5E-01	
02825	argininosuccinate lyase	53	7			X	172.4	2.1E-21	X
04347	aspartate kinase	66	4			X	2.0	3.4E-01	
00649	tryptophan synthase	77	4			X	-1.1	7.3E-01	
04156	homoserine kinase	38	2			X	48.2	5.3E-02	
	**Glucose metabolism**								
00057	fructose-bisphosphatase	38	4	X			-270.6	2.1E-02	
00697	UDP-glucose epimerase	41	2	X			4.8	1.1E-01	
02664	D-lactate dehydrogenase	58	3		X		5.7	2.9E-02	
01820	pyruvate kinase	57	8			X	4.0	4.4E-06	

04969	UDP-glucose dehydrogenase	51	6			**X**	4.0	2.9E-02	
06770	fructose-bisphosphate aldolase	39	5			**X**	-1.0	9.0E-01	
06699	glyceraldehyde-3-phosphate dehydrogenase	36	4			**X**	-1.1	8.5E-01	
06900	phosphoglycerate mutase	59	3			**X**	-3.9	1.3E-01	
06313	phosphoglucomutase	62	2			**X**	2.9	3.0E-01	
**Pentose phosphate pathway**									
03245	glucose-6-phosphate 1-dehydrogenase	57	6			**X**	6.1	2.3E-03	
01984	transaldolase	35	6			**X**	-1.5	3.5E-01	
07561	phosphogluconate dehydrogenase	54	5			**X**	6.2	1.4E-05	
**Pyrimidine, purine metabolism**									
03335	ribose-phosphate diphosphokinase	36	2	**X**			1.8	4.3E-01	
00143	phosphoribosylamidoimidazole-succinocarboxamide synthase	90	8		**X**		-1.9	3.8E-02	
07502	CTP synthase	70	7		**X**		2.7	6.6E-02	
00700	bifunctional purine biosynthesis protein PURH	65	13			**X**	9.0	2.6E-16	
02858	adenylosuccinate synthase	47	7			**X**	2.1	5.0E-02	
00441	IMP dehydrogenase	58	5			**X**	24.8	4.0E-03	X
01877	GMP synthase	60	2			**X**	1.3	7.2E-01	
02794	dihydroorotate dehydrogenase	57	2			**X**	-3.3	5.4E-01	
**Other cell metabolism proteins**									
01745	sn-glycerol-3-phosphate dehydrogenase NAD	42	3	**X**			-1.7	5.8E-01	
01528	serine hydrolase, ovarian cancer-associated gene 2	33	2	**X**			1.2	8.6E-01	
04687	stearoyl-CoA 9-desaturase	66	2	**X**			3.0	3.2E-01	
06001	phosphomevalonate kinase	59	2	**X**			1.6	7.1E-01	
00345	porphobilinogen synthase	36	2	**X**			2.5	5.6E-01	
06908	pyridoxine biosynthesis protein	35	6		**X**		2.9	1.6E-02	
04605	C-3 sterol dehydrogenase	49	2		**X**		37.6	1.3E-03	
01577	glutamate dehydrogenase	49	12			**X**	12.1	4.4E-27	
05292	alpha,alpha-trehalose-phosphate synthase	74	4			**X**	-9.3	6.4E-08	X
02918	acetyl-CoA C-acetyltransferase	42	3			**X**	8.4	8.3E-02	
03322	UDP-xylose synthase	47	2			**X**	18.8	2.3E-03	X
06535	Ribosomal L1 domain containing protein	39	2			**X**	8.7	1.8E-01	
06421	acetolactate synthase	39	2			**X**	-3.1	3.5E-01	
01102	oxidoreductase	33	4			**X**	-30.6	4.6E-02	
**Proteins involved in cell trafficking and membrane fusion**									
01031	Yop1, ER associated Ypt Interacting Protein.	41	3	**X**			-6.5	4.8E-01	
01211	An epsilon-COPI subunit of the coatomer, Sec28	33	2	**X**			-1.7	4.7E-01	
04194	protein transport protein Sec13	36	4		**X**		-1.2	5.5E-01	
00565	Vps1	77	6			**X**	-1.8	8.1E-02	
03853	small COPII coat GTPase SAR1	21	2			**X**	1.5	7.9E-01	
01274	coatomer subunit gamma, Sec21	101	2			**X**	-3.2	2.1E-01	
**Proteins related to heat shock proteins**									
01696	DNAj protein	58	2	**X**			-5.8	3.3E-01	
00305	chaperone activator	40	5		**X**		-3.0	2.4E-03	X
03891	heat shock protein	61	22			**X**	2.3	3.0E-13	X
07347	heat shock protein	100	21			**X**	-166.8	6.5E-27	X
01404	hsp71-like protein	64	11			**X**	-1.2	8.9E-02	
06208	heat shock protein	86	10			**X**	-5.7	8.8E-11	X
03347	chaperone	92	7			**X**	-10.6	1.3E-05	X
01750	chaperone	70	6			**X**	-2.0	0.0E+0	X
07346	t-complex protein 1	61	6			**X**	1.7	2.4E-01	
04976	Ribosome associated DnaJ chaperone Zuotin	51	5			**X**	3.1	2.1E-02	
06106	chaperone regulator	37	3			**X**	-3.2	2.8E-01	
07558	wos2 protein	25	2			**X**	-2.2	7.2E-01	

**Proteins related to proteins synthesis**									
01548	eIF4A homologue	45	3	**X**			2.9	1.3E-01	
02677	Tma46 homologue	40	2		**X**		-1.1	9.2E-01	
00413	2OG-Fe(II) oxygenase Tpa1	74	2		**X**		-2.0	5.0E-01	
02657	eIF3 subunit G	32	4		**X**		1.2	6.6E-01	
06563	eIF3 subunit EifCf	33	4			**X**	1.6	6.8E-01	
06061	eIF3 subunit 6	52	3			**X**	2.6	6.1E-02	
02507	eIF3 subunit 3	41	2			**X**	-1.1	9.6E-01	
00509	eIF3 subunit M	49	4			**X**	3.1	3.4E-04	X
02486	eIF2 beta subunit	36	5			**X**	143.4	1.9E-05	X
01833	eIF4E homologue	33	4			**X**	3.8	1.4E-03	X
04628	eIF6	26	2			**X**	1.8	6.8E-01	
02948	Polypeptide release factor	54	4			**X**	6.5	1.3E-02	
01270	W2 domain-containing protein,	49	3			**X**	56.8	1.3E-02	
00754	Rli1 homologue	68	4			**X**	36.9	5.4E-06	X
**Proteins related to proteasome**									
07719	26S protease regulatory subunit	47	3	**X**			6.2	2.1E-01	
06153	26S protease regulatory subunit	52	4		**X**		7.4	1.6E-02	
04666	26S protease regulatory subunit 6B	45	2		**X**		-1.0	9.9E-01	
00062	26S proteasome non-ATPase regulatory subunit 3	59	8			**X**	4.0	4.5E-04	X
02239	26S proteasome regulatory subunit 4	49	3			**X**	12.5	2.1E-04	X
**Other**									
02672	RNA binding protein Nmd3	63	3	**X**			10.9	1.2E-01	
06742	C2H2-type zinc finger protein	46	3	**X**			42.9	5.0E-02	
00108	RuvB-like helicase 1	52	2	**X**			4.5	2.7E-01	
03541	protein with the PP2C domain	41	2	**X**			1.3	7.3E-01	
03584	U5 snRNP-specific protein	40	2	**X**			2.5	4.2E-01	
05091	conserved hypothetical protein	23	2	**X**			4.8	5.0E-01	
03863	histone H4 arginine methyltransferase RmtA	39	5		**X**		1.9	2.7E-01	
04948	tubulin beta chain	49	4		**X**		2.8	8.0E-02	
01780	Drg1 homologue	41	4		**X**		3.9	1.1E-01	
01136	conserved hypothetical protein	38	4		**X**		4.5	1.1E-01	
07637	LSU ribosomal protein L3P	34	2		**X**		1.3	7.4E-01	
02880	YchF homologue	50	8			**X**	1.4	3.0E-01	
02943	Slm1 homologue	61	5			**X**	-32.4	2.5E-07	X
06746	histone h2b	15	4			**X**	2.4	5.4E-03	X
04028	RNA binding protein	39	4			**X**	1.2	7.4E-01	
06633	40s ribosomal protein s15	17	3			**X**	1.3	2.2E-01	
01361	importin alpha subunit	59	3			**X**	10.0	3.1E-04	X
03052	PP2Cc protein phosphatase	55	3			**X**	-2.2	1.6E-01	
07352	PP2C protein phosphatase	32	3			**X**	-1.3	6.2E-01	
01770	MRS7 family protein	75	3			**X**	-4.1	3.6E-02	
02585	RfeF	51	3			**X**	1.5	3.3E-01	
03249	mRNA export protein Mlo3	22	3			**X**	-1.3	8.9E-01	
05827	tRNA binding protein	41	3			**X**	2.1	5.7E-01	
04209	voltage-gated potassium channel beta-2 subunit	40	3			**X**	-31.3	3.5E-01	
02754	40S ribosomal protein S12	16	2			**X**	3.3	9.0E-03	X
03627	cyclophilin A	17	2			**X**	-8.7	2.1E-01	
04439	vacuolar ATP synthase	57	2			**X**	21.2	3.6E-02	
00626	conserved hypothetical protein	24	2			**X**	-5.2	2.0E-02	

* A CNAG number based on the *C. neoformans* H99 database (Broad Institute)

* *A negative number indicates a fold change increase in binding at 37°C.
